# Inability to pursue nonrigid motion produces instability of spatial perception

**DOI:** 10.1126/sciadv.adp6204

**Published:** 2024-11-06

**Authors:** Krischan Koerfer, Tamara Watson, Markus Lappe

**Affiliations:** ^1^ Institute for Psychology and Otto Creutzfeldt Center for Cognitive and Behavioral Neuroscience, University of Münster, Münster 48149, Germany.; ^2^ School of Social Sciences and MARCS Institute for Brain, Behaviour and Development, Western Sydney University, Sydney, NSW 2751, Australia.

## Abstract

Vision generates a stable representation of space by combining retinal input with internal predictions about the visual consequences of eye movements. We report a type of nonrigid motion that disrupts the connection between eye movements and perception, causing visual instability. This motion is accurately perceived during fixation, but it cannot be pursued. Catch-up saccades are accurately directed to the moving target but the motion stimulus appears to jump in space with each saccade. Our results reveal four major findings about perception and the visuomotor system: (i) Pursuit fails for certain types of motion; (ii) pursuit and catch-up saccades are independently controlled; (iii) prediction of saccade consequences is independent from saccade control; and (iv) the visual stability of moving objects relies on similar motion mechanisms as pursuit.

## Teaser

A visual motion that cannot be pursued causes a breakdown of perceptual stability across saccades.

## INTRODUCTION

Because of the structure of the retina with a small fovea centralis of high resolution, humans perform eye movements to gather information from the visual world and track moving objects. These eye movements, in turn, produce self-generated changes in the retinal input. Predictive mechanisms in the visuomotor system estimate the expected retinal consequences of each eye movement and ensure a stable perception of the world despite the disarray on the retina (*1*, *2*). This prediction becomes more challenging if objects move in the world. Saccadic eye movements, the fastest changes of gaze, take about 100 ms to plan and 50 to 80 ms to execute. Therefore, to accurately reach a moving object with a saccade, the visual system has to estimate the movement of the object during saccade planning and execution and calculate the landing point of the saccade so that it reaches the position where the object will be once the saccade has arrived (*3*). Prediction of the object’s motion is also essential to perceive the object as continuous and not abruptly jumping in the visual scene (*4*–*6*) because motion perception is briefly suppressed during saccades (*7*).

Continuous tracking of a moving object is achieved by a gradual movement of the eye called smooth pursuit. While the tracked object is kept stationary on the fovea, the image of the background is moving across the retina as the eye moves over the scene. Yet, same as for saccades, the visual world appears largely stable during pursuit despite the induced motion of the image across the retina (*8*). Undertaking smooth pursuit relies on the motion signal from the object (*9*). For speeds up to 30°/s, the smooth pursuit gain, i.e., the ratio of eye speed to object speed, is close to unity. If the gain is lower, then small so-called catch-up saccades supplement the tracking to keep the target on the fovea. This interplay of smooth and saccadic pursuit is controlled by a shared neural system that ensures accurate interaction with moving objects and a stable perception of our environment (*10*, *11*). In the present study, we report on a new class of motion stimuli that break pursuit and induce failures of trans-saccadic visual stability.

## RESULTS

### A motion that can be perceived but not pursued

**Fig. 1.**
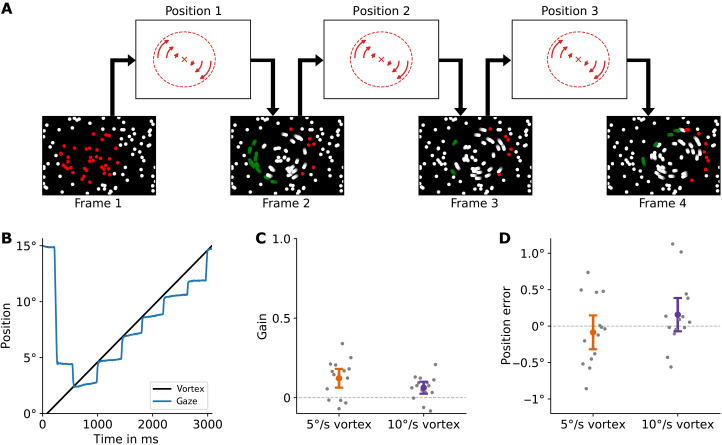
A nonrigid motion stimulus for which smooth pursuit eye movement, a hallmark of primate oculomotor control, fails and which can be tracked only by saccadic eye movements. (**A**) Stimulus consists of a field of dots over which, in sequential frames, a vortex pattern is moved. Between successive frames, the dots are moved according to the vortex rotation. The position of the vortex moves to the right over time. As the vortex position moves, new dots at the leading edge are picked up in the next frame (shown in red for illustration purposes), and dots at the trailing edge are dropped (green) and return to the static background. Examples of the stimulus can be seen in movies S1 and S2. (**B**) Example eye-tracking data of one participant trying to pursue the vortex’s leading edge shows tracking by saccades and little movement in the smooth phases between saccades. (**C**) Average pursuit gain of smooth pursuit is very low across participants (*n* = 15). (**D**) Average error of the catch-up saccades is close to zero, showing that they land successfully on the moving vortex target. Negative values correspond to landing behind, and positive values correspond to landing in front of the vortex’s leading edge.

Many ambient motions in the environment are characterized by the movement of a pattern across a field of particles. Consider, for example, wind blowing across a field of grass or a body of water. While the individual elements overall remain in place, the wave travels across in a perceivable way. To study this type of motion perception, we have recently created a novel stimulus that presents the movement of a motion pattern across a field of elements as its sole motion cue (*12*). It consists of a rotating vortex that moves across a field of dots (Fig. 1A). In a frame-by-frame animation of this pattern, each single frame displays the same random dot distribution that is perturbed by the vortex. Across frames, the dots within the vortex are moved according to the vortex motion pattern, i.e., their position is rotated around the center position. Over time, the center position and the area over which the motion pattern is applied shift so that the vortex moves across the dot field. Crucially, the dots are not moved along with the vortex. Instead, new dots are picked up at the leading edge, and old dots are dropped at the trailing edge as the vortex moves. Therefore, the movement of the vortex is independent of the movement of the dots. Observers were able to accurately judge the direction, speed, and trajectory of the vortex’s motion (*12*). For an example, see movie S1.

However, when we asked observers to track the moving vortex with a smooth movement of their eyes, we found that they were unable to do so (experiment 1, compare movie S2). We asked 15 participants to pursue the leading edge of a vortex, 3.75° in diameter, moving horizontally from left to right with either 5° or 10°/s. Participants followed the stimulus with an alteration of saccades and periods in which the eye is practically static (Fig. 1B). The smooth pursuit gain in these periods was nearly zero (Fig. 1C), a deficiency counterbalanced by frequent catch-up saccades. For the slow-moving vortex, the gain was 0.12 ± 0.06, and for the fast-moving vortex, the gain was 0.06 ± 0.04. Usually, pursuit at these speeds is highly efficient. For example, fig. S2 (D, H, and I) shows that pursuit gain was 0.798 ± 0.127 and 0.785 ± 0.178 for an equally fast, rigidly moving control stimulus. The data from the nonrigid vortex hence show that smooth pursuit of the vortex is nonviable at either of the tested speeds. Instead, participants tracked the moving vortex with a sequence of saccades instead of a smooth pursuit movement.

### Pursuit is not possible but saccades are accurate

We then analyzed the quality of the saccades that the participants directed to the moving vortex. Figure 1D illustrates the horizontal error of the saccade, with positive values indicating a position ahead of the vortex’s leading edge and negative values indicating a position trailing the vortex’s leading edge. The saccade error was −0.09 ± 0.23 degrees of visual angle (dva) for the slow-moving vortex and 0.16 ± 0.23 dva for the fast-moving vortex, both not significantly different from 0 (*P* = 0.48 and *P* = 0.20). This indicates a high degree of accuracy in saccade targeting during ongoing motion. In addition, the measured saccade landing positions were significantly ahead of the approximated vortex position at saccade planning (fig. S1). Hence, the saccade targeting efficiently used the motion of the vortex to predict its position after saccade latency and saccade duration.

Therefore, the vortex’s motion provokes a substantial failure in smooth pursuit, while catch-up saccades maintain their accuracy. This reveals a pronounced dissociation between the saccade and the pursuit systems for this unique motion type.

### Futile pursuit leads to a failure of visual stability

**Fig. 2.**
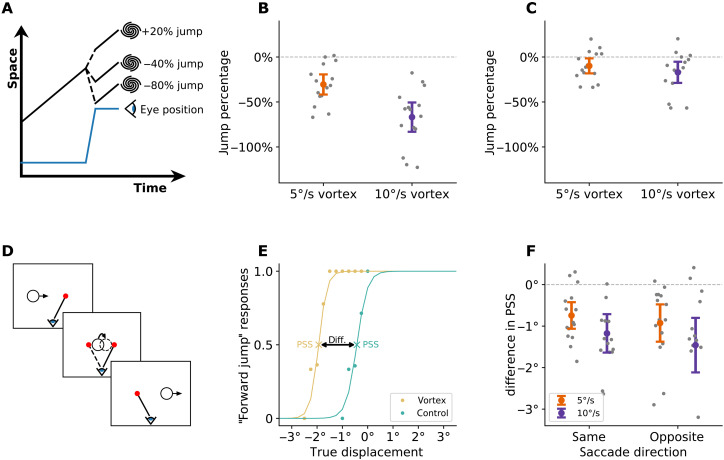
The vortex appears to jump during saccades indicating a breakdown of trans-saccadic visual stability, an essential process of visual perception. (**A**) In the jump size adjustment task, participants could adjust a physical displacement of the moving vortex during each saccade such that the movement appeared smooth. (**B**) At 0%, the vortex was moving smoothly across the screen without any physical displacement. In contrast, the chosen jump percentages show that the vortex was perceived as smooth when it was displaced substantially backward on each saccade. (**C**) In a subsequent replay condition, participants saw the movement of the vortex, including their previously chosen jump percentage, while fixating on a stationary target. During fixation, the jump percentage was readjusted close to zero, implying smooth perception when no jumps were present. (**D**) In experiment 3, participants made saccades between two stationary targets (red dots), while the vortex moved across the screen. The vortex physically jumped forward or backward during the saccade, and participants had to discriminate the direction of the jump. (**E**) Psychometric curve (yellow) of one participant for saccade direction opposite to target motion at 10°/s. Comparison data (blue) are for a rigidly moving object at the same speed. Points of subjective stability (PSSs) mark the true displacements at which the motions appear perceptually smooth. The two curves are offset indicating that the vortex had to be displaced by 1.5° during each saccade to appear as smooth as a rigid motion. (**F**) Differences in PSS between rigid control and the vortex. The data show a significant shift of the PSS for the vortex. The shift is larger for higher speeds but does not depend on saccade direction.

Viewing the stimulus, we also noted that, when attempting to pursue the vortex, the vortex movement appeared unstable and jumpy, pointing toward a partial loss of visual stability (as the reader can observe in movie S2). These apparent jumps did not seem to occur when one fixated on a stationary target while viewing the moving vortex (as in movie S1). Thus, the perceived jumps seemed to be related to the attempted pursuit or the saccades made during the attempted pursuit. To investigate visual stability during attempted pursuit, in experiment 2, we asked 15 participants to pursue the vortex but physically jumped the vortex during each saccade. In this experiment, each catch-up saccade triggered a physical positional jump of the vortex. The size of this jump equaled a percentage of the amplitude of the catch-up saccade (Fig. 2A). Participants were given control over the jump size and asked to adjust it until they perceived the vortex motion as smooth. Each trial started initially with a physical jump size of zero. Participants could then adjust the jump size via small-positive or -negative increments by button press while the stimulus looped across the display. When a participant perceived the vortex motion as smooth, they stopped the trial via a different button press.

We found that the movement of the vortex was perceived as smooth if it physically jumped backward on each saccade (Fig. 2B). The vortex moving at 5°/s had to jump 30.5 ± 11.2% of the saccade size backward to be perceived as moving smoothly. The vortex moving at 10°/s had to jump backward by 66.9 ± 16.3%. Both values are significantly different from zero (*P* < 0.001) and also from each other (*P* < 0.001). This emphasizes a severe loss of the ability to perceive a stable motion of the vortex across saccades.

To ensure that the failure of this trans-saccadic visual stability was indeed associated with the attempted pursuit, we replayed recordings of vortex movement, including the adjusted jumps, in a separate experimental session while participants fixated. Consistent with the earlier observation that, during fixation a regular and continuous movement of the vortex appeared as smooth, when we replayed the previously recorded trials, in which the vortex physically jumped according to the recorded catch-up saccades in the pursuit condition, participants correctly perceived it as jumping (movie S3). We then asked the participants to readjust the jump size until the movement appeared smooth to the fixating eye. In each trial, the jump size started with the average jump size that the participant had set as smooth in the pursuit condition. We found that participants readjusted their former chosen jump size close to zero and significantly different from the pursuit condition (−9.9 ± 8.3%, *P* = 0.007 for the slow-moving vortex and −17.0 ± 11.8%, *P* = 0.00004 for the fast-moving vortex; Fig. 2C). This emphasizes that there is no issue with visual stability during fixation and that the loss of visual stability during attempted pursuit is caused by the catch-up saccades.

### Breakdown of visual stability occurs also with saccades from fixation and is independent from saccade direction

In the above experiments, saccade direction, size, and duration were always coupled with the vortex motion. Catch-up saccades were always in the direction of the vortex motion and faster vortex motion necessitated larger and longer catch-up saccades. To disentangle the impact of saccade properties and vortex motion on the failure of visual stability, in experiment 3, we presented the vortex motion while participants made saccades of controlled size and direction between two stationary points, 3.75° apart (Fig. 2D). Participants were instructed to fixate on the first red dot and initiate a saccade toward a second red dot once it appeared and then fixate on it for the rest of the trial. The vortex moved across the screen and, during the saccade between the two fixation points, jumped either forward or backward. Participants had to report the direction of the jump. Jump size was varied by a staircase procedure to determine psychometric curves for jump direction discrimination. Saccade direction was either in the same or opposite direction of the vortex motion. Unlike in the previous experiments, in this experiment, saccades did not occur within the context of pursuit because the participants initially fixated on one of the stationary dots.

For comparison, participants performed the same task with a rigid motion control stimulus. In this stimulus dots moved as if glued to a rigid, nonrotating disk the same size as the vortex that traveled across the screen, thereby occluding dots in the background (fig. S2D). Figure 2E shows an example of the resulting psychometric curves of one participant for vortex and rigid control moving with 10°/s and saccade direction opposite to target motion direction. In this example, the point of subjective stability (PSS), i.e., the point at which the participant’s rating of jump direction was at chance level, differed by −1.5° between the vortex and the rigid control. Thus, the vortex appeared most stable when it physically jumped backward by 1.5° during the saccade. Across participants, the average difference in PSS between vortex and control was negative, i.e., the vortex appeared stable when it physically jumped backward during the saccade in all tested conditions (Fig. 2F; all *P* < 0.001).

Notably, the PSS difference, and hence the optimal jump size for smooth motion, scaled with the speed of the vortex movement (Wilcoxon signed-rank test, *P* = 0.007) but was independent of saccade direction (Wilcoxon signed-rank test, *P* = 0.82). These results emphasize that the loss of visual stability does not depend on the saccade properties but on the movement of the vortex during the saccade.

### During assisted pursuit, smooth pursuit and saccades are well integrated and visual stability is restored

**Fig. 3.**
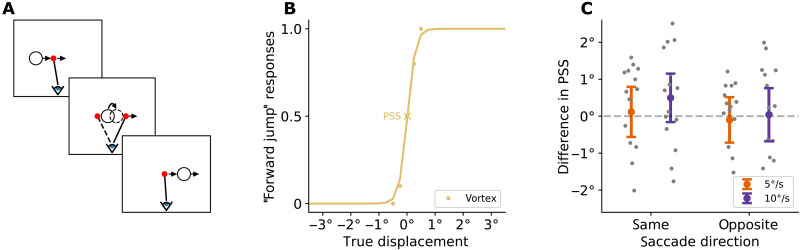
Trans-saccadic visual stability of the vortex can be restored if smooth pursuit is aided by the movement signal of a single dot moving along with the vortex. (**A**) Participants smoothly pursued a red dot that moved along with the vortex and then made a saccade to a second red dot that suddenly appeared. They had to detect a physical jump of the vortex during the saccade. (**B**) Example psychometric curve for saccade direction opposite to target motion at 10°/s. (**C**) Difference between the points of subjective equality for the vortex during pursuit and the rigid control stimulus from experiment 3 during fixation was around zero in all conditions, indicating that the discrimination was similarly good for both vortex and rigid control.

In experiment 4, we investigated whether visual stability can be restored if the pursuit and saccade systems are brought into alignment again. We added a red dot to the stimulus display that was placed to the left or to the right of the vortex and moved alongside the vortex (Fig. 3A). Fifteen participants, the same as in experiment 3, pursued the red dot. They were able to do this with a high average gain of 0.80 ± 0.08 and 0.79 ± 0.06 for velocities of 5 and 10°/s, respectively (fig. S3). Thus, the motion of their eyes matched the motion of the vortex as if they were able to pursue it. This allowed us to establish the combination of two conditions being investigated in this experiment: First, a motion of the vortex on the retina that would occur if participants were able to pursue and, second, an oculomotor situation in which pursuit and saccades function together normally. As in experiment 3, participants had to make saccades toward a second red dot, which also moved along the vortex. The vortex was physically jumped during the saccade, and participants had to discriminate the direction of the jump. Results showed that their PSSs were not different from those for the rigid motion during fixation (data from experiment 3) for either speed and saccade direction (all *P* > 0.1) (Fig. 3C). Thus, when the saccade and pursuit system are both functional and well integrated, there is no loss of visual stability for the vortex motion.

In this experiment, the red dots served as a motion signal to assist the pursuit. We also tested whether such a signal would allow to jump-start the pursuit of the vortex, and whether sustained pursuit might be possible if the red dot were dropped during the pursuit. This was not the case, however, as pursuit gain quickly plummeted once the red dot disappeared (fig. S4).

### The movement of the vortex can be used only by selected systems

Our results alert us to several unexpected dissociations in the perceptual and motor systems. First, the movement of the vortex can be perceived but not pursued. This indicates a dissociation between motion perception and smooth pursuit. Second, while smooth pursuit cannot track the vortex’s motion, the saccades land accurately on the vortex position. This indicates a dissociation between smooth and saccadic aspects of pursuit. Third, while saccades land accurately on the vortex, the vortex has to jump backward from that position to be perceived as smoothly moving. This indicates a dissociation between prediction for motor control and prediction for visual stability. The loss of trans-saccadic visual stability for moving objects also indicates a dissociation of two components of perception, namely, perception during fixation and smooth pursuit phases and perception across saccades, emphasizing distinct perceptual mechanisms of these components. When we realigned pursuit and saccade systems, these dissociations disappeared, and visual stability was restored.

**Fig. 4.**
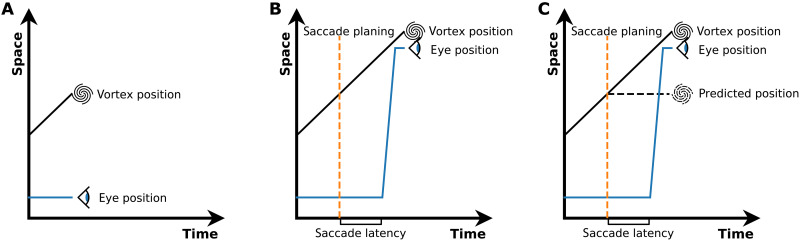
The vortex movement is accessible only by subsystems of perception and control. The corresponding model is illustrated by three space-time diagrams across the phases of attempted pursuit. (**A**) The pursuit system cannot use the motion signal of the vortex, and gaze does not follow the vortex’s motion. (**B**) As a positional difference between gaze and the vortex accumulates over time, a catch-up saccade is initiated. The saccade system can predict the movement of the vortex during saccade planning and execution and brings gaze accurately to the vortex’s location. (**C**) The visual system, however, cannot predict the motion of the vortex. Hence, trans-saccadic prediction for visual stability fails to incorporate the movement of the vortex. Therefore, when the actual position of the vortex is detected after the saccade, it is perceived as a sudden jump from the incorrectly predicted position to the actual position. The size of the jump is determined by the distance the vortex has traveled during saccade latency and saccade duration.

We believe that these dissociations can be explained if we assume that only selected subsystems of the visual and oculomotor systems have access to the movement of the vortex. Figure 4 offers a space-time diagram of the model to illustrate how these assumptions account for our findings. First, as the movement of the vortex cannot be used for pursuit, Fig. 4A depicts that pursuit gain is zero and gaze is not following the vortex. Second, at some point in time, the distance between gaze and the vortex becomes so large that a catch-up saccade is prepared (Fig. 4B). This preparation requires prediction because the vortex continues to move, while the saccade is planned and executed. Because the saccades did not land on the vortex position at saccade planning but landed accurately on the vortex after the saccade (fig. S1), the saccadic system can use the movement signal of the vortex to predict and accurately target the vortex’s future position. Third, for perceptual stability, the visual system also has to predict the position of the vortex after the saccade. To this end, it has to account for the displacement due to the eye movement and for the vortex’s movement during saccade latency and execution. We propose that, in contrast to the accurate prediction of the saccadic system, the movement of the vortex cannot be integrated into this trans-saccadic prediction for perception. Thus, the ongoing motion of the vortex during saccade latency and execution is not added to the prediction of its post-saccadic position, as if the vortex remained stationary during that time (Fig. 4C). Then, upon completion of the saccade, the vortex is observed in its actual position, producing a mismatch with its predicted position and a perceived jump from the predicted to the observed position.

In this model, the perceived jump size is the product of vortex speed with the sum of the saccade latency (the planning phase) and the saccade duration (the execution phase), as described in the model section in Materials and Methods. To evaluate whether the proposed explanation is consistent with the recorded data, we fitted the adjusted jump sizes displayed in Fig. 2B and the difference in PSS between vortex and rigid control displayed in Fig. 2F using the saccadic latency as the only free parameter. For each participant, we calculated in each experiment and for each speed the latency that fitted the observed jump size. We then performed an analysis of variance (ANOVA) to determine whether pooling data across experiments and speeds is feasible. A two-way ANOVA showed no effect of experiment (*P* = 0.69), vortex speed (*P* = 0.94), or their interaction (*P* = 0.85), indicating that saccade latencies did not depend on the experiment or speed. Hence, the data were pooled across experiments and speed conditions to determine the best fitting mean saccadic latency for each participant. From that, the mean saccade latency and the 95% confidence interval across participants were calculated on the basis of the variance across participants. This resulted in an estimated average saccade latency of 111 ± 24 ms, which is well in alignment with typical latencies of catch-up saccades (*13*). Thus, the data from both experiments can be well explained by the proposed model and a biologically plausible saccade latency.

## DISCUSSION

Using a novel visual motion stimulus that captures properties of nonrigid motion, we found previously unidentified dissociations between perception and smooth pursuit eye movement, between pursuit and saccadic eye movement, and between prediction for action and prediction for the perceptual stability of the visual world. These dissociations require a re-evaluation of our understanding of the interactions of the visual and oculomotor systems and the brain areas involved.

It has long been known that smooth pursuit is not possible without a motion percept (*14*). The vortex motion studied here produces a clear motion precept but does not provide an appropriate signal for smooth pursuit. Smooth pursuit and motion perception are normally closely linked (*15*, *16*), but pursuit does not always follow perception. For example, the direction of pursuit might sometimes diverge from the direction of the motion percept (*17*, *18*). Our results show that a particular class of nonrigid motion fails to access the pursuit system altogether. Because the motion signal for pursuit is supplied by dedicated brain areas, the middle temporal (MT) and medial superior temporal (MST) visual areas (*9*), we predict that the vortex motion signal is not present in these areas. This may seem puzzling because area MST is known to be specifically sensitive to motion patterns such as rotations (*19*), which are the basis for the vortex motion. However, the motion signal of the vortex is not caused by the rotation itself but by the translation of the center of rotation over time. Area MST is blind to changes of motion patterns (*20*). Thus it may be that area MST, although responsive to the pattern of the vortex, is not responsive to the changes of its position. Moreover, by comparing local and global disturbances of the vortex pattern, we have previously shown that the information carrying the vortex motion is obtained not from the global pattern of rotation but from local differential motion, known as curl (*12*). The absence of curl sensitivity in MT and MST (*21*) is therefore consistent with the pursuit inability and suggests that the perception of the vortex motion relies on different brain areas. Many areas of the brain respond to visual motion (*22*). Candidate areas that may support perception of the vortex motion could be, for example, the kinetic occipital (KO) area (*23*) or the visual area 6 (V6) (*24*). Area KO is known to be strongly activated by motion borders (*25*), and motion borders evoke strong local curl in the motion field. The findings of the present study are not restricted to vortex motion but can be experienced with other types of nonrigid motions as well and even with a simple motion border (fig. S2).

While the findings of this study generalize to a variety of motion patterns and remain stable across different parameters, the percept and oculomotor behavior are distinct from other higher order motion stimuli. Higher-order motion perception entails several kinds of second order motion, among them theta motion (*26*), which, similar to our stimuli, consists of motion defined by a motion border that moves. However, unlike our vortex motion, theta motion and other second order motions can be pursued [fig. S5A; (*27*–*30*)], albeit with reduced gain compared to first-order motion, and do not perceptually jump during pursuit (fig. S5B).

Often when we track a moving object it is with a combination of smooth pursuit and saccades (*11*). In a successful tracking task, intercepting saccades, catch-up saccades, and step-back saccades bring the tracked object close to the fovea, and horizontal, vertical, and torsional components of smooth pursuit minimize the relative motion on the retina (*31*, *32*). Smooth pursuit was long considered driven by velocity error and saccades by position error (*33*–*35*). Recent research, however, has emphasized links between both control processes (*11*, *36*) and between their neural mechanisms (*10*, *37*, *38*). Thus, the combination of pursuit and saccades for tracking a moving object is now considered a shared control system. Our results show that saccades during tracking have access to the vortex motion signal, but smooth pursuit does not. Thus, there must be a level of separation between the saccade and smooth pursuit control. Previous research has shown that the direction of saccades and pursuit can dissociate (*39*, *40*). Our study shows that even in the total breakdown of smooth pursuit, saccades during attempted pursuit are accurately directed at the target. Hence, the motion of the vortex can be used to predict the vortex position for saccade planning, and the vortex motion signal must be available in the corresponding neuronal pathway. Central to the control of tracking by smooth pursuit and saccades is the frontal eye field (FEF), in which neurons share a representation of both speed and position error (*37*). Because smooth pursuit is not possible for the vortex motion, its motion signal is unlikely represented in FEF. Instead, the prediction of the vortex position for saccade control might be provided by the lateral intraparietal area (LIP) which, in interaction with the superior colliculus (SC), is vital for predicting the position of a moving target for interceptive saccades (*3*, *41*, *42*). Our finding that the saccades landed accurately on the vortex edge suggests that the vortex motion signal might be available in area LIP.

Prediction of the future position of objects across a saccade is crucial for the perceptual stability of the world during eye movements (*2*, *43*, *44*). For the vortex motion, this stability is broken as the vortex seems to jump with every saccade. In previous studies, conditions that break visual stability were achieved through brief artificial interferences with the natural visual input during or before a saccade (*7*, *45*, *46*). While these studies have shown the fragility of visual stability around saccade preparation and performance, our study shows that breaks of visual stability can occur during continuous stimulus presentation and without artificial interference.

In interpreting these findings, it is important to distinguish two distinct components of the trans-saccadic visual stability for moving objects. First, the change of visual input due to the eye movement has to be accounted for, i.e., the internally generated motion of the visual scene (*2*, *43*). Second, the change of visual input due to the eigenmotion of any moving object during saccade execution has to be accounted for, i.e., the externally generated motion (*47*, *48*). Only this second component of visual stability is impaired for the moving vortex. One might be tempted to think that a signal that accurately leads the saccade to the position of the moving vortex, perhaps in area LIP, would also accurately predict the vortex position for perception, but this was not the case in our data. Therefore, the perceptual prediction depends on different pathways than the saccade prediction. This observation might be better understood if one considers that such a prediction would have to be made for any object moving in the visual field, not only for the moving target of the catch-up saccade, and that each moving object could have a different direction and velocity. We, thus, propose that the areas that integrate the eigenmotion of each object across saccades for perceptual stability differ from the area that integrates the motion of the saccade target and that these areas have no access to the vortex motion signal. A distinct perceptual mechanism and brain area can then also explain why the trans-saccadic visual stability is impaired, while perception during fixation is possible.

The inability to predict the vortex motion across saccades for trans-saccadic stability explains the perceived jumpiness during attempted pursuit, which necessitates frequent catch-up saccades, and during saccades between two stationary points. However, it also raises questions about the smoothness of perception during fixation, when fixational microsaccades occur. While the frequency and size of fixational saccades can vary among individuals and tasks, they are usually significantly smaller and shorter in duration than saccades (*49*), thus having a lesser impact on visual stability. Yet, some residual jumpiness of the vortex and other similar motion patterns during fixation is expected and reflected in the data presented in fig. S6.

Given the degradation of both smooth pursuit and perception that we have outlined, it is natural to wonder about the likelihood of needing to pursue a stimulus like this in a real-world scenario. The carefully constructed stimulus has enabled us to find several unexpected dissociations between perception and action that would be concerning for real-world perceptual tasks. However, while the stimulus is inspired by the movement of substances such as smoke or water, it purposefully does not contain all the possible visual cues that this stimuli would but only those cues that are specific to this type of motion and set it apart from rigid-object motion. When looking at the environmental stimuli that inspired our stimulus, it is likely that there are several other motion cues, same as in the assisted tracking of experiment 4, supporting both pursuit and perception. It is only by carefully ensuring that these are not present that we have been able to uncover separation between the perception and ocular motor systems.

Our results highlight the impressive level of integration occurring between different perceptual and motor processes. The dissociation we have shown between motion perception, visual stability, and ocular motor control of different eye movements emphasizes the complex interaction of potentially discreet processes routinely occurring to support effortlessly stable perception and accurate interaction with the world. As they all become apparent using a single type of motion stimulus, this stimulus might provide opportunities for further investigation of the combination of signals in the visual and oculomotor systems and the underlying neuronal pathways.

## MATERIALS AND METHODS

### All experiments

Stimuli were presented on an Eizo FlexScan F930 monitor with 1152 × 870 pixels and 75-Hz refresh rate in a dimmed room. Participants rested their head on a custom chin rest, 67 cm in front of the screen, resulting in 30.89° × 23.32° visual angle. Each frame of the stimulus consisted of 10,000 pixel-sized white dots on a black background. The vortex had a diameter of 3.75° and rotated with 45°/s. The disk in the rigid control condition was of the same size but did not rotate. Eye-tracking data were recorded with an Eyelink 1000 (SR Research, Ontario, Canada). Calibration was performed with a white nine-point grid on a black background. The experimental procedure was performed with MATLAB (MathWorks, Natick, MA) using the Psychophysics Toolbox. Saccade detection was done with the Eyelink software. The study was approved by the Ethics Committee of the Department of Psychology and Sport Science of the Universität Münster, Münster, Germany (approval number 2017-01-ML). All participants gave written informed consent to participate in the study and to their data being stored before the experiments. The data were stored anonymously to ensure participant confidentiality and data protection.

### Experiment 1

Fifteen participants (nine female and six male) with age ranging from 20 to 36 years were instructed to pursue the leading edge of the vortex. The vortex moved with 5 or 10°/s from left to right. The vortex center started at 0.25 of the total screen width and ended at 0.75 of the total screen width, ensuring that the vortex was entirely visible at all times. Stimulus duration was 3 and 1.5 s, respectively. There were 10 repetitions per velocity. The last frame of the stimulus was shown until participants started a new trial via button press. The gain was calculated by accumulating the times and position changes of the gaze direction between saccades, starting from the first saccade after stimulus onset and up to the last saccade before trial end. Phases between saccades in which the eye tracking signal was partly lost due to blinks etc. were discarded. Dividing accumulated position change by accumulated time allowed to calculate average pursuit speed and pursuit gain for every participant and velocity condition. The 95% confidence intervals were calculated using the variance across the 15 participants. Position error of catch-up saccades was calculated by the difference of the detected saccade landing position and the position of the vortex edge for all saccades except the first and last saccade of each trial. The 95% confidence intervals were calculated using the variance across the 15 participants.

### Experiment 2

Experiment 2 displayed the same vortex motion as experiment 1, with the difference that the vortex automatically restarted from the left after reaching the right end point creating a continuously looping trial. Fifteen participants (seven female and eight male, age 19 to 38) were instructed to pursue the leading edge of the vortex and to adjust the jump size until they perceived the vortex as moving smoothly, i.e., with as little jumps as possible. Every time a saccade end was detected, the vortex was immediately displaced by a percentage of the saccade length depending on the current jump size setting. Unknown to the participants, the jump size setting started at 0% each trial. It could be adjusted continuously by pressing the left or right arrow key, which increased/decreased the percentage in 20% increments up to a maximum of ±140% of saccade length. Once participants were satisfied with the current state of their jump size setting, they were instructed to observe a full passage of the vortex from left to right three times, first with the jump setting one increment higher than their preliminary chosen setting, then one increment lower, and lastly returning to their preliminary chosen setting. Only if participants perceived the vortex as moving smoother with their preliminary chosen jump size setting compared to the other two, or if they could not tell the difference, they logged in their choice by pressing the space bar. The chosen jump size was saved, and the movement of the vortex in the last passage, including all jumps, was saved for the second part of the experiment. Then, the next trial was started. The vortex moved at speeds of 5 or 10°/s. The velocity conditions were in random order with 10 repetitions each. Because of continuously looping trials, the duration of the experiment varied between 10 and 50 min.

In the second part of experiment 2, participants were instructed to fixate on a red fixation cross (16 × 16 pixels) in the center of the screen. In each trial, a recorded movement of the vortex from part 1 was replayed in a loop and the participants again had to adjust the jump size until the movement appeared smooth during fixation. Instructions were identical to part 1 of the experiment. The trials started with the formerly chosen jump size, and the jumps of the vortex were scaled by the ratio of former chosen and current jump size accordingly. For every participant, the mean chosen jump percentage was calculated per speed and pursuit/fixation condition, and the 95% confidence intervals were calculated on the basis of the variance of the 15 participants.

### Experiment 3

Experiment 3 used the same vortex motion as experiments 1 and 2 and an additional rigid control stimulus. In the rigid control stimulus, the vortex was replaced with a black disk with the same diameter as the vortex. Fixed to the disk were white dots, the same dot size and density as the background. When the disk moved, it occluded the background. In addition, there could be red fixation dots (10 pixels diameter) 1.875° left and right from the center. The experiment was split into two blocks. In one block, the left dot would be present at the beginning of the trial. Once the vortex reached the center, the red dot on the right appeared and both red dots were presented until the end of the trial. In the other block, the order of appearance was swapped with the right dot being displayed at the beginning and the left appearing in the middle of the trial. The vortex moved from left to right in both blocks. The order of blocks was random for each participant. The blocks were separated by a break, a repetition of the instructions, and a new calibration. Fifteen participants (seven female and eight male, age 19 to 38) were instructed to initially fixate the red dot and then make a saccade to the second red dot once it appeared and to fixate it until the end of the trial. As soon as a saccade onset was detected after the display of the second dot, the vortex would be displaced. The displacement was controlled by a staircase procedure with 25 instances starting at 0° displacement and with ±0.25° increments.

Participants reported whether they perceived a jump of the vortex to the right (right arrow key) or left (left arrow key). The response was possible as soon as the jump was displayed. If the response was provided before stimulus end, then the stimulus would still be displayed until the end and the next trial would start automatically afterward. Otherwise, the last frame of the stimulus was shown until a response had been made. The displacement of the vortex in the next trial was adjusted by 0.25° to the right if the response was the left arrow key and by 0.25° to the left if the response was the right arrow key. There were two repetitions of the staircase procedure for each of the velocity conditions (5 and 10°/s), both stimulus types (vortex and rigid control) and for the two blocks (saccades from left to right and saccades from right to left), resulting in 2 × 25 × 2 × 2 × 2 = 400 trials in total per participant. The order of the 16 staircases was random for each participant. Data from 15 participants were collected. For each combination of velocity, stimulus type, and saccade direction conditions, the responses in the two staircases were used to calculate the right and left response ratios for every displacement value. These response ratios were then fitted with a psychometric function of the form 1/{1 + exp[−(*x* − *a*)/*b*]}. The optimal coefficient *a* defined the PSS. For both velocity conditions and both saccade directions, the difference of the PSSs between vortex and rigid control conditions were calculated for every participant. The 95% confidence intervals were calculated on the basis of the variance across the 15 participants. For each participant, the PSS differences in same and opposite condition were combined, and a two-sample Wilcoxon signed-rank test was performed to analyze the impact of the speed of the vortex on the differences of PSSs.

### Experiment 4

Experiment 4 was done on the same day as experiment 3 and with the same participants. The experimental design was identical to that of experiment 3 with the following two exceptions. First, only the vortex stimulus was used. Second, the red dots were centered around the initial vortex position (1.875° left and right from the vortex center) and moved with the same speed and direction as the vortex. Participants were instructed to pursue the red dot to make a saccade toward the other red dot as soon as it appeared and to pursue it afterward. The red dots were not displaced together with the vortex once the saccade was detected but instead moved on smoothly. Feedback and staircase procedures were identical to experiment 3. The PSS was calculated for all four combinations of both speeds and saccade directions as in experiment 3. For every participant, the difference of the PSS to that of the rigid condition in experiment 3 was calculated for each of the four parameter combinations. The 95% confidence intervals were calculated on the basis of the variance across the 15 participants.

### Model

The model assumes that the perceived jump of the vortex across a saccade is caused by the movement of the vortex during saccade latency and saccade execution: *s*_j_ = *v_v_ ·* (*t*_l_ + *t*_d_), with *s*_j_ denoting the size of the jump, *v*_v_ denoting the velocity of the vortex, and *t*_l_ and *t*_d_ denoting the saccade latency and saccade duration, respectively. Given *s*_j_, *v*_v_, and *t*_d_, the saccade latency can be computed as *t*_l_ = (*s*_j_ − *v*_v_ · *t*_d_)*/v*_v_. For experiment 2, the jump sizes of the vortex and the saccade durations during each last passage of the vortex before participants logged their response were averaged. Then, the saccade latency was calculated for each participant, each speed, and every repetition. Afterward, the saccade latencies were averaged across repetitions, resulting in one saccade latency per participant and vortex speed for experiment 2. For experiment 3, for each participant and both speeds, the difference in the PSS was used as *s*_j_, and the saccade durations of the corresponding trials were averaged. With these values, one saccade latency per participant and vortex speed was calculated for experiment 3.

A two-way ANOVA with independent samples was performed to test for the impact of experiment type, vortex speed, or their interaction on saccade latency. Given no significant impact of either experiment or vortex speed, saccade latencies of both speeds were averaged per participant, and the combined data of both experiments were used to calculate the mean saccade latency and the 95% confidence interval based on the variance across participants.

## Supplementary Materials

### The PDF file includes:

Figs. S1 to S6 Legends for movies S1 to S7 References
