# Supplementary material for: Inability to pursue nonrigid motion produces instability of spatial perception
Source: Sci Adv. 2024 Nov 6;10(45):eadp6204. doi: 10.1126/sciadv.adp6204 (PMC11540027; doi:10.1126/sciadv.adp6204)
Supplement: Supplementary file 1 — Figs. S1 to S6 Legends for movies S1 to S7 References [file sciadv.adp6204_sm.pdf]

Science Advances
AAAS

Supplementary Materials for

Inability to pursue nonrigid motion produces instability of spatial perception

Krischan Koerfer *et al.*

Corresponding author: Krischan Koerfer, krischan.koerfer@uni-muenster.de

Sci. Adv. **10**, eadp6204 (2024)
DOI: 10.1126/sciadv.adp6204

The PDF file includes:

Figs. S1 to S6
Legends for movies S1 to S7
References

Other Supplementary Material for this manuscript includes the following:

Movies S1 to S7

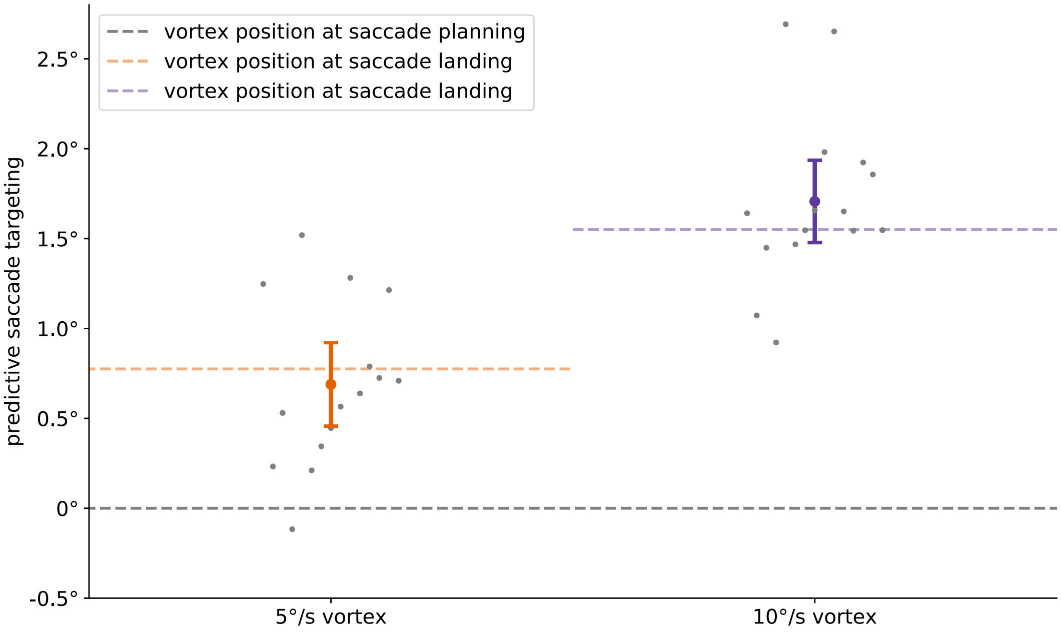

Fig. S1.

In Experiment 1, we investigated if the saccade system can accurately target the moving vortex. In the additional analysis presented here, we approximated the vortex position at the time of saccade planning by assuming an average catch-up saccade latency of 125ms (13). We then determined how far ahead the catch-up saccade landed from that position. The figure shows the distance of the saccade landing positions to the approximated vortex position at saccade planning. For comparison, the orange and purple lines indicate the average vortex position at the time of saccade landing for slow and fast vortex speed, respectively. The data shows that the saccade system targeted the saccade significantly ahead of the approximated vortex position at saccade planning (p-values <0.0001 for both speeds) and close to the actual vortex position at the time of saccade landing. This shows again that the saccade systems can use the motion of the vortex to predict and target the vortex position at saccade landing.

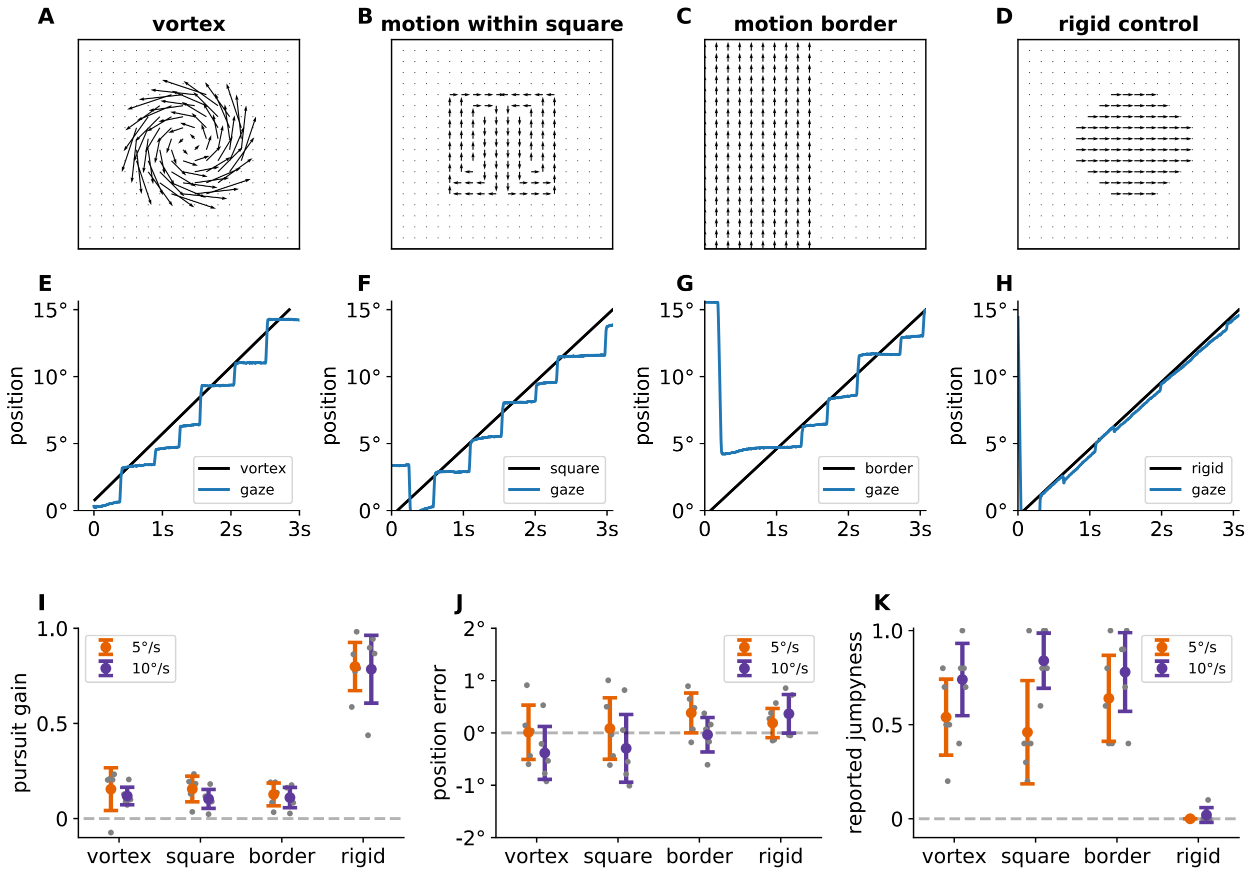

Fig. S2.
Our findings are not specific to rotational motion but generalize to other moving patterns. Five participants were asked to pursue each of four different motion patterns and report whether the pattern appeared smooth or jumpy. (**A**) Replication of the vortex motion experiment. (**B**) Complex motion pattern within a traveling square: in contrast to the vortex, this is not a global rotation pattern and it has no simple correspondence in the natural environment, but it also has local curl. (**C**) Simple motion border in which dots move downwards once they are picked-up by the traveling border. The motion border produces local curl as well. (**D**) Rigid control stimulus. In this stimulus, all dots moved as if attached to a disk that moved across the screen, covering and uncovering the background dots during the movement. Local curl is present at the edges of the disk, but importantly this is not the only motion cue, there are also first-order motion and occlusion cues. (**E-G**) Example eye tracking data of one participant showing that of none of the non-rigid stimuli allowed smooth pursuit. This was compensated by frequent and accurate catch-up saccades. (**H**) The rigid control can be pursued with high efficiency. (**I**) Average pursuit gain across participants for the different stimuli. Error bars indicate 95% confidence intervals. (**J**) Mean error of catch-up saccades. Saccades were accurate for all stimuli. (**K**) Report on jumpiness. All three moving motion patterns are significantly more often perceived as jumping than the rigid control. Higher reported rate for faster movement re-emphasize that the jump is driven by the movement of the pattern across the saccades. We conclude that the failure of pursuit and the failure of visual stability do not rely on the global rotation pattern of the vortex. Yet, all three motion patterns in a-c would stimulate local curl detectors.

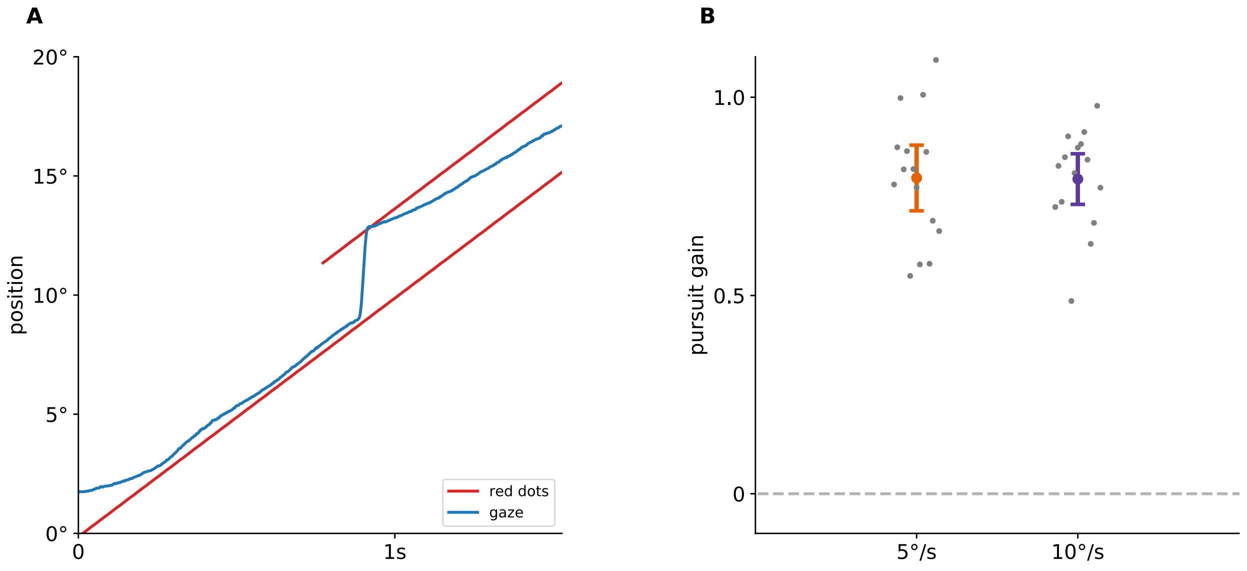

Fig. S3.

In Experiment 4, a red dot was added to the display that moved along with the vortex. Participants could pursue the dot successfully. Panel (**A**) shows example eye tracking data of one trial with speed of $10^{\circ}/s$. Panel (**B**) shows pursuit gain across the 15 participants. Colored points and bars show the mean gain, which was 0.796 ± 0.083 for a speed of $5^{\circ}/s$ and 0.794 ± 0.064 for a speed of $10^{\circ}/s$. Grey points show gains for the individual participants.

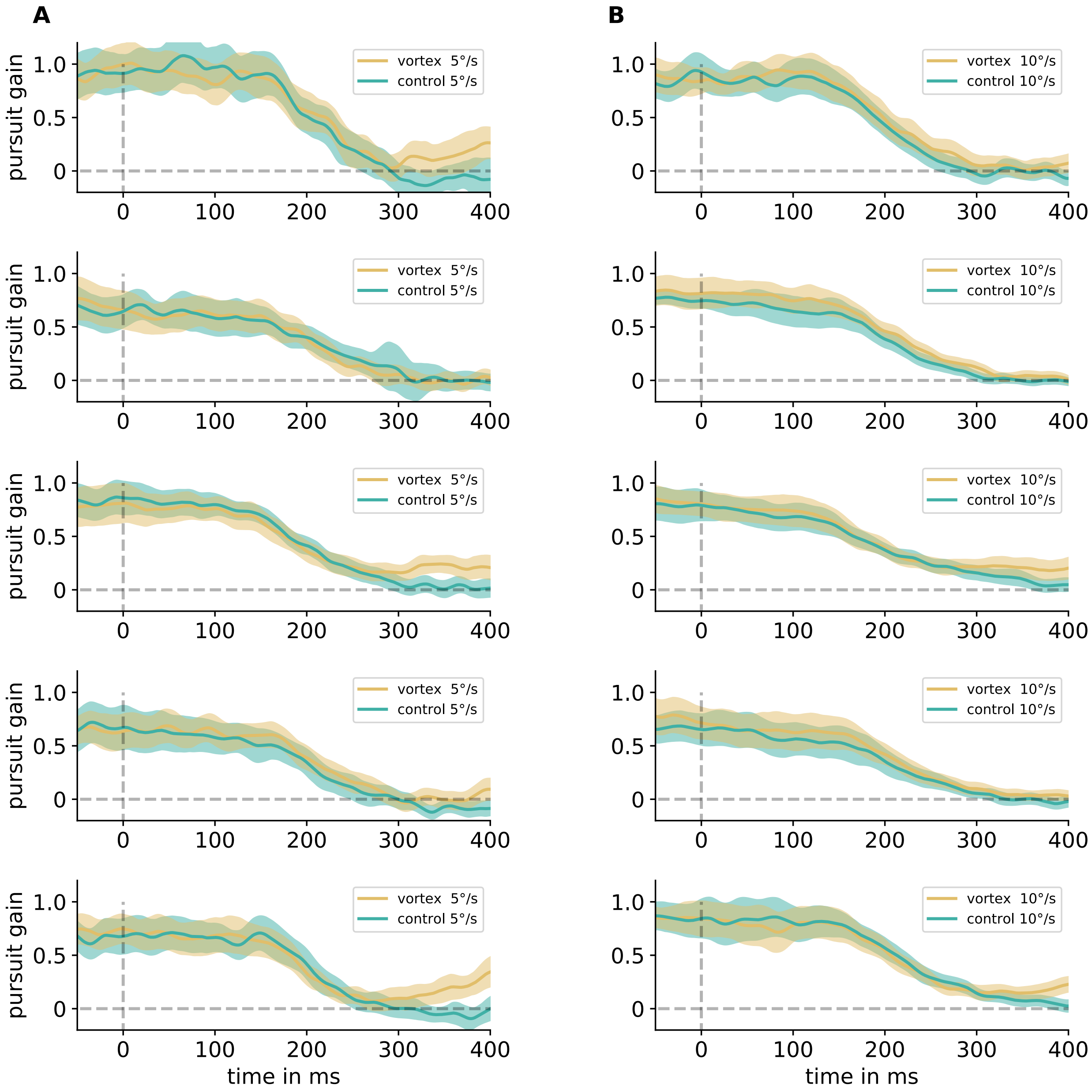

Fig. S4.

In an additional experiment similar to Experiment 4, the red dot that supported pursuit disappeared halfway during each trial. The figure shows the average pursuit gain after the red dot disappeared ($t = 0$) for 100 trials of five participants. Column (**A**) shows the decline in pursuit gain after the red dot disappeared for the speed of $5^{\circ}/s$, column (**B**) shows the decline for $10^{\circ}/s$. In the control condition, the vortex as well as the red dot disappeared halfway during the trial leaving just a dotted background. For all participants, the pursuit declines similarly for both conditions. For the vortex condition, the pursuit gain does not decline to 0 but stabilizes at a small positive gain for some participants. This is consistent with the measured pursuit gains of the vortex in Experiment 1. In conclusion, even if pursuit was successfully initiated it could not be sustained just by the movement of the vortex.

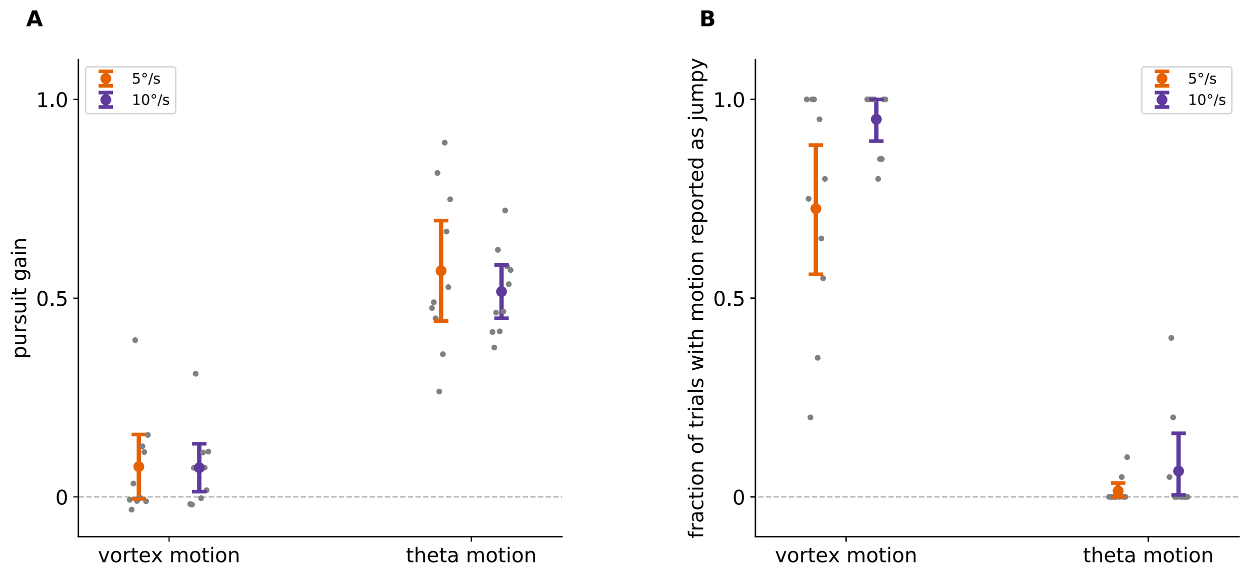

Fig. S5.

In a further control experiment, we compared oculomotor behavior and perception of our vortex stimulus with that of second-order motion, exemplified by theta motion (26). Theta motion shares some of the properties of the vortex motion. It is also a higher order motion that is defined by the movement of first order motion: a window moves across a background with random dot texture, within the window an equal random dot texture is moved in opposite direction of the window's movement (26). Thus, the first order component of the theta motion is in opposite direction of its second order component. Likewise, every single frame of the theta motion stimulus, being a random distribution of pixel luminance, is devoid of structural information which is also true for the vortex motion. However, there are also differences between the two stimuli. For the vortex, the movement of a motion pattern, which we propose is processed by a curl based mechanism, is the only motion cue (12). Theta motion activates this curl based mechanism as well but importantly, it is not the only cue. In theta motion there is a moving window in which the motion is presented and the background is occluded by the moving texture/dots within the window. In the vortex stimulus dots of the background that are initially static are picked-up by the moving vortex preventing any occlusion cues.

Previous work on the pursuit of theta motion showed that pursuit onset latency is impaired for theta motion and pursuit speed is somewhat reduced compared to first-order motion (28). Yet pursuit capability in general is retained, consistent with other second order motions at the speed of our stimuli (27, 28, 30, 50). While these findings appear distinct from the breakdown of pursuit capability for the vortex motion, given the similarities of stimulus properties of theta motion and the vortex motion we wanted to investigate the differences in oculomotor behavior and perception in a direct comparison. We presented trials with either vortex motion or theta motion at speeds of 5°/s or 10°/s and instructed ten participants to follow the objects with their gaze. After each trial, participants had to rate the motion as either "smooth" or "jumpy". Panel (**A**) shows the average pursuit gain. With a gain of 0.08 ± 0.08 and 0.07 ± 0.06, pursuit was again close to impossible for the vortex motion. Pursuit gain was significantly higher for the theta motion: 0.57 ± 0.13 for a speed of 5°/s and 0.52 ± 0.07 for a speed of 10°/s (two-way repeated measures ANOVA F-value = 194). Panel (**B**) shows the fraction of trials in which participants rated the motion as "jumpy"

for both stimulus conditions and velocities. For the vortex, the motion was perceived as jumpy in a large fraction of trials for both velocities: 0.73 (+0.16, -0.17) and 0.95 (+0.05, -0.05). In contrast, the theta motion was almost never perceived as jumpy: 0.02 (+0.02, -0.02) and 0.06 (+0.07, -0.06). Thus, the percept was quite different, with the vortex motion being perceived as significantly jumpier (two-way repeated measures ANOVA F-value = 205). The difference may result from the additional occlusion cue that is present in theta motion but not in the vortex stimulus. The results of this experiment emphasize that the vortex motion presented in this study is distinct from other second-order motion both in terms of perception as well as oculomotor behavior.

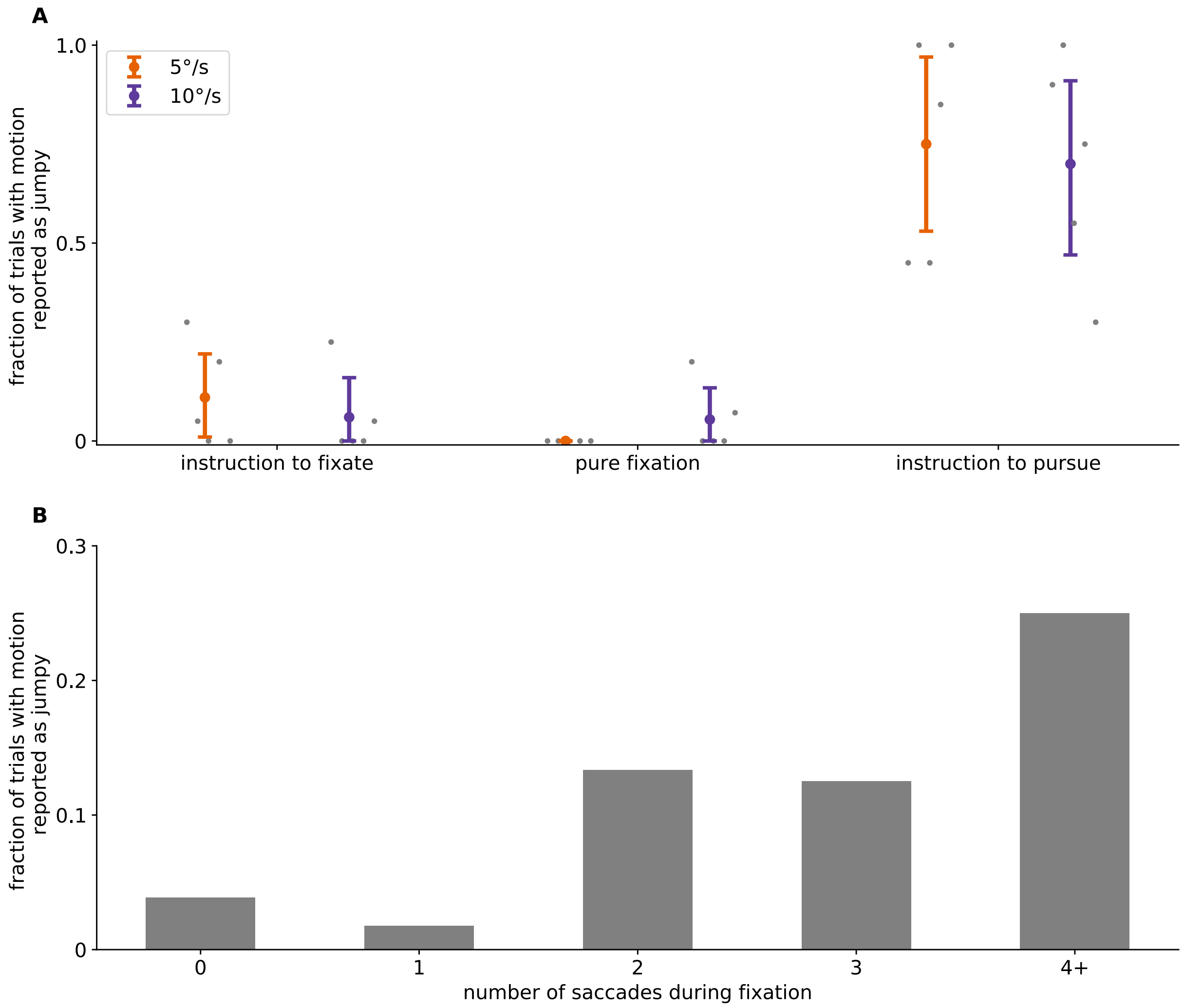

Fig. S6.

For the vortex stimulus, a difference between fixation and pursuit is that during fixation, background dots remain stationary on the retina. In an additional experiment, we tested whether the perceived jumpiness during attempted pursuit relies on the motion onset of the background points. For this experiment we created a stimulus that also had motion in the background points. We used a motion border similar to that depicted in Fig. S2C, but with dots moving upward on the left side of the motion border and downward on the right side, or vice versa. This ensured that, regardless of eye movement, all dots moved across the retina in both viewing conditions. Five participants were instructed to fixate on a red dot or to follow the motion border with their gaze. After each trial, participants rated the motion as either "smooth" or "jumpy." (**A**) The fraction of trials reported as jumpy for both speed and instruction conditions. During fixation, participants perceived the motion border as mostly smooth, with just 0.11 (-0.10, +0.11) and 0.06 (-0.06, +0.10) of trials rated as jumpy. During pursuit, the movement was rated as jumpy in 0.75 (-0.22, +0.22) and 0.70 (-0.22, +0.21) of trials, respectively. Thus, the loss of visual stability was significantly reduced during fixation, although some residual jumpiness remained. This residual jumpiness during fixation was resulting from difficulties to fixate, which were expected

since the stimulus contained strong motion cues in the up/down direction. The data labeled as "pure fixation" used only trials from the fixation instruction in which at most one saccade occurred. This reduced the fraction of trials that were reported as jumpy further, for the slow moving motion border to 100% smooth percept for all participants and trials. (**B**) The relationship between the number of trials with motion reported as jumpy and the number of saccades made during fixation. There is a clear trend: with more saccades, the motion is reported as jumpy in more trials. Thus, the movement of a motion pattern is perceived as mostly smooth during fixation, and the residual jumpiness is driven by fixational saccades. This supports our model, which suggests that the movement of a motion pattern cannot be accurately predicted across saccades, leading to a loss of trans-saccadic stability.

Movie M1.

These videos present demonstrations of the main effects described in the paper. To avoid issues with video compression in the MP4 format, the dot size in these demonstrations was set to 3 by 3 pixels and the total dot number was reduced to 5000.
Video 1 shows the basic vortex stimulus as it moves across the screen with a fixation cross. When one fixates on the cross the smooth movement of the vortex can be seen.

Movie M2.

Video 2 presents the same smooth vortex movement without a fixation cross and an instruction to pursue. The vortex appears to jump and move irregularly during attempted pursuit.

Movie M3.

Video 3 presents a result of the jump adjustment task that was also used during the replay condition. In the jump adjustment task, the participant adjusted a physical jump of the vortex during each catch-up saccade so that the vortex appeared smooth. Presented to the fixating eye, the same jumps are clearly visible.

Movie M4.

Video 4 presents the stimulus "motion within square" that is analyzed in supplementary Fig. S2.

Movie M5.

Video 5 presents the stimulus "motion border" that is analyzed in supplementary Fig. S2.

Movie M6.

Video 6 presents the stimulus "rigid control" that is analyzed in supplementary Fig. S2.

Movie M7.

Video 7 presents a motion border stimulus similar to M5, but with dots moving upward on one side and downward on the other. The stimulus is described and analyzed in supplementary Fig. S6.

REFERENCES AND NOTES

1. H. von Helmholtz, *Handbuch der Physiologischen Optik* (Leopold Voss, 1896).

2. R. H. Wurtz, W. M. Joiner, R. A. Berman, Neuronal mechanisms for visual stability: Progress and problems. *Phil. Trans. R. Soc. Lond. B Biol. Sci.* **366**, 492–503 (2011).

3. J. Churan, A. Kaminiarz, J. C. B. Schwenk, F. Bremmer, Coding of interceptive saccades in parietal cortex of macaque monkeys. *Brain Struct. Funct.* **226**, 2707–2723 (2021).

4. V. Gysen, P. De Graef, K. Verfaillie, Detection of intrasaccadic displacements and depth rotations of moving objects. *Vision Res.* **42**, 367–391 (2002).

5. A. Fracasso, A. Caramazza, D. Melcher, Continuous perception of motion and shape across saccadic eye movements. *J. Vis.* **10**,14 (2010).

6. M. Szinte, P. Cavanagh, Spatiotopic apparent motion reveals local variations in space constancy. *J. Vis.* **11**, 4 (2011).

7. J. Ross, M. C. Morrone, M. E. Goldberg, D. C. Burr, Changes in visual perception at the time of saccades. *Trends Neurosci.* **24**, 113–121 (2001).

8. W. Filehne, Über das optische Wahrnehmen von Bewegungen. *Zeitschrift für Sinnesphysiology* **53**, 134–145 (1922).

9. S. G. Lisberger, Visual guidance of smooth-pursuit eye movements: sensation, action, and what happens in between. *Neuron* **66**, 477–491 (2010).

10. R. J. Krauzlis, Recasting the smooth pursuit eye movement system. *J. Neurophysiol.* **91**, 591–603 (2004).

11. A. Goettker, K. R. Gegenfurtner, A change in perspective: The interaction of saccadic and pursuit eye movements in oculomotor control and perception. *Vision Res.* **188**, 283–296 (2021).

12. K. Koerfer, M. Lappe, Perceived movement of nonrigid motion patterns. *PNAS Nexus* **1**, pgac088 (2022).

13. S. De Brouwer, D. Yuksel, G. Blohm, M. Missal, P. Lefèvre, What triggers catch-up saccades during visual tracking? *J. Neurophysiol.* **87**, 1646–1650 (2002).

14. M. Hashiba, T. Matsuoka, S. Baba, S. Watanabe, Non-visually induced smooth pursuit eye movements using sinusoidal target motion. *Acta Otolaryngol. Suppl.* **116**, 158–162 (1996).

15. M. Spering, A. Montagnini, Do we track what we see? Common versus independent processing for motion perception and smooth pursuit eye movements: A review. *Vision Res.* **51**, 836–852 (2011).

16. P. Kreyenmeier, R. Kumbhani, J. A. Movshon, M. Spering, Shared mechanisms drive ocular following and motion perception, *eNeuro* **11**, ENEURO.0204–24.2024 (2024).

17. A. E. Hughes, Dissociation between perception and smooth pursuit eye movements in speed judgments of moving Gabor targets. *J. Vis.* **18**, 4 (2018).

18. M. Spering, M. Pomplun, M. Carrasco, Tracking without perceiving: A dissociation between eye movements and motion perception. *Psychol. Sci.* **22**, 216–225 (2011).

19. K. Tanaka, H. A. Saito, Analysis of motion of the visual field by direction, expansion/contraction, and rotation cells clustered in the dorsal part of the medial superior temporal area of the macaque monkey. *J. Neurophysiol.* **62**, 626–641 (1989).

20. M. Paolini, C. Distler, F. Bremmer, M. Lappe, K. P. Hoffmann, Responses to continuously changing optic flow in area MST. *J. Neurophysiol.* **84**, 730–743 (2000).

21. G. A. Orban, L. Lagae, A. Verri, S. Raiguel, D. Xiao, H. Maes, V. Torre, First-order analysis of optical flow in monkey brain. *Proc. Natl. Acad. Sci. U.S.A.* **89**, 2595–2599 (1992).

22. P. Dupont, G. A. Orban, B. De Bruyn, A. Verbruggen, L. Mortelmans, Many areas in the human brain respond to visual motion. *J. Neurophysiol.* **72**, 1420–1424 (1994).

23. P. Dupont, B. De Bruyn, R. Vandenberghe, A. M. Rosier, J. Michiels, G. Marchal, L. Mortelmans, G. A. Orban, The kinetic occipital region in human visual cortex. *Cereb. Cortex* **7**, 283–292 (1997).

24. S. Pitzalis, M. I. Sereno, G. Committeri, P. Fattori, G. Galati, F. Patria, C. Galletti, Human V6: The medial motion area. *Cereb. Cortex* **20**, 411–424 (2009).

25. S. Van Oostende, S. Sunaert, P. Van Hecke, G. Marchal, G. A. Orban, The kinetic occipital (KO) region in man: An fMRI study. *Cereb. Cortex* **7**, 690–701 (1997).

26. J. M. Zanker, Theta motion: A paradoxical stimulus to explore higher order motion extraction. *Vision Res.* **33**, 553–569 (1993).

27. F. Butzer, U. Ilg, J. Zanker, Smooth-pursuit eye movements elicited by first-order and second-order motion. *Exp. Brain Res.* **115**, 61–70 (1997).

28. T. Miyamoto, K. Miura, T. Kizuka, S. Ono, Properties of smooth pursuit and visual motion reaction time to second-order motion stimuli. *PLOS ONE* **15**, e0243430 (2020).

29. T. Miyamoto, K. Miura, T. Kizuka, S. Ono, The effect of explicit cues on smooth pursuit termination. *Vision Res.* **189**, 27–32 (2021).

30. M. J. Hawken, K. R. Gegenfurtner, Pursuit eye movements to second-order motion targets. *J. Opt. Soc. Am. A Opt. Image Sci. Vis.* **18**, 2282–2296 (2001).

31. P. M. Daye, G. Blohm, P. Lefèvre, Catch-up saccades in head-unrestrained conditions reveal that saccade amplitude is corrected using an internal model of target movement. *J. Vis.* **14**, 12 (2014).

32. J. Edinger, D. Pai, M. Spering, Coordinated control of three-dimensional components of smooth pursuit to rotating and translating textures. *Invest. Ophthalmol. Vis. Sci.* **58**, 698–707 (2017).

33. C. Rashbass, The relationship between saccadic and smooth tracking eye movements. *J. Physiol.* **159**, 326–338 (1961).

34. D. A. Robinson, The mechanics of human saccadic eye movement. *J. Physiol.* **174**, 245–264 (1964).

35. E. Kowler, S. P. McKee, Sensitivity of smooth eye movement to small differences in target velocity. *Vision Res.* **27**, 993–1015 (1987).

36. J.-J. Orban de Xivry, P. Lefevre, Saccades and pursuit: two outcomes of a single sensorimotor process. *J. Physiol.* **584**, 11–23 (2007).

37. D. Liston, R. J. Krauzlis, Shared response preparation for pursuit and saccadic eye movements. *J. Neurosci.* **23**, 11305–11314 (2003).

38. J.-J. Orban de Xivry, S. J. Bennett, P. Lefèvre, G. R. Barnes, Evidence for synergy between saccades and smooth pursuit during transient target disappearance. *J. Neurophysiol.* **95**, 418–427 (2006).

39. M. R. Maechler, N. H. Heller, M. Lisi, P. Cavanagh, P. U. Tse, Smooth pursuit operates over perceived not physical positions of the double-drift stimulus. *J. Vision* **21**, 6 (2021).

40. M. Lisi, P. Cavanagh, Different extrapolation of moving object locations in perception, smooth pursuit, and saccades. *J. Vision* **24**, 9 (2024).

41. R. J. Krauzlis, L. Goffart, Z. M. Hafed, Neuronal control of fixation and fixational eye movements. *Philos. Trans. R Soc. B Biol. Sci.* **372**, 20160205 (2017).

42. E. N. Eskandar, J. A. Assad, Distinct nature of directional signals among parietal cortical areas during visual guidance. *J Neurophysiol.* **88**, 1777–1790 (2002).

43. D. Melcher, C. L. Colby, Trans-saccadic perception. *Trends Cogn. Sci.* **12**, 466–473 (2008).

44. P. Cavanagh, A. R. Hunt, A. Afraz, M. Rolfs, Visual stability based on remapping of attention pointers. *Trends Cogn. Sci.* **14**, 147–153 (2010).

45. L. Matin, D. G. Pearce, Visual perception of direction for stimuli flashed during voluntary saccadic eye movements. *Science* **148**, 1485–1488 (1965).

46. B. Bridgeman, D. Hendry, L. Stark, Failure to detect displacement of the visual world during saccadic eye movements. *Vision Res.* **15**, 719–722 (1975).

47. S. K. Miura, M. Scanziani, Distinguishing externally from saccade-induced motion in visual cortex. *Nature* **610**, 135–142 (2022).

48. C. Lee, J. Lee, Visual motion perception at the time of saccadic eye movements and its relation to spatial mislocalization. *Ann. N. Y. Acad. Sci.* **1039**, 160–165 (2005).

49. M. Rucci, M. Poletti, Control and functions of fixational eye movements. *Annu. Rev. Vis. Sci.* **1**, 499–518 (2015).

50. A. Lindner, U. Ilg, Initiation of smooth-pursuit eye movements to first-order and second-order motion stimuli. *Exp. Brain Res.* **133**, 450–456 (2000).
